# Pulmonary Histoplasmosis, Taiwan, 1997–2024

**DOI:** 10.3201/eid3209.260437

**Published:** 2026-09

**Authors:** Chih-Wei Liang, Chieh Yu Hsieh, Shang-Yi Lin

**Affiliations:** Division of Infectious Diseases, Kaohsiung Medical University Hospital, Kaohsiung Medical University, Kaohsiung, Taiwan (C.-W. Liang, S.-Y. Lin); Kaohsiung Municipal Siaogang Hospital, Kaohsiung (C.-Y. Hsieh)

**Keywords:** histoplasmosis, dimorphic fungi, fungi, *Histoplasma capsulatum*, non-endemic regions, pulmonary mycosis, Taiwan

**To the Editor:** We read with interest about the cases of *Histoplasma capsulatum* infection in Taiwan, described by Kao et al. (*1*). However, molecular characterization of clinical isolates from Taiwan remains limited.

We report a case of disseminated histoplasmosis diagnosed in southern Taiwan in 2024. A 57-year-old man had persistent fever, weight loss, and progressive dyspnea. He had no history of diabetes, autoimmune disease, immunosuppressive therapy, or HIV infection. Imaging revealed diffuse pulmonary infiltrates and hepatosplenomegaly. Laboratory tests showed pancytopenia, hyperferritinemia, hypertriglyceridemia, and elevated liver enzymes, raising suspicion for hemophagocytic lymphohistiocytosis. Bone marrow examination demonstrated intracellular yeast forms within histiocytes, and metagenomic next-generation sequencing detected *H. capsulatum* fungi. The patient received liposomal amphotericin B (3 mg/kg/d) for 10 days, followed by oral itraconazole (200 mg 2×/d), with gradual clinical improvement. A bone marrow fungal culture yielded *H. capsulatum* fungi 28 days after sample collection. The patient never traveled to histoplasmosis-endemic regions, but he did engage in vegetable gardening on farmland in suburban Kaohsiung, where bat activity has been documented, although he reported no direct contact with bat guano, bird roosts, or imported animals.

Our phylogenetic analysis showed that the isolate clustered within the Latin American A1 lineage, together with reference strains from Mexico and Honduras (Figure). That finding was unexpected because most Asia isolates belong to the Eurasian clade (*2*). Possible explanations include imported infection, historical introduction, or unrecognized environmental reservoirs. Because *H. capsulatum* fungi commonly inhabit soil enriched with bat guano or bird droppings, suitable ecological niches might exist in subtropical Taiwan (*2*–*4*). Our findings suggest that lineage diversity of *H. capsulatum* fungi circulating in Asia might be underestimated.

**Figure:**
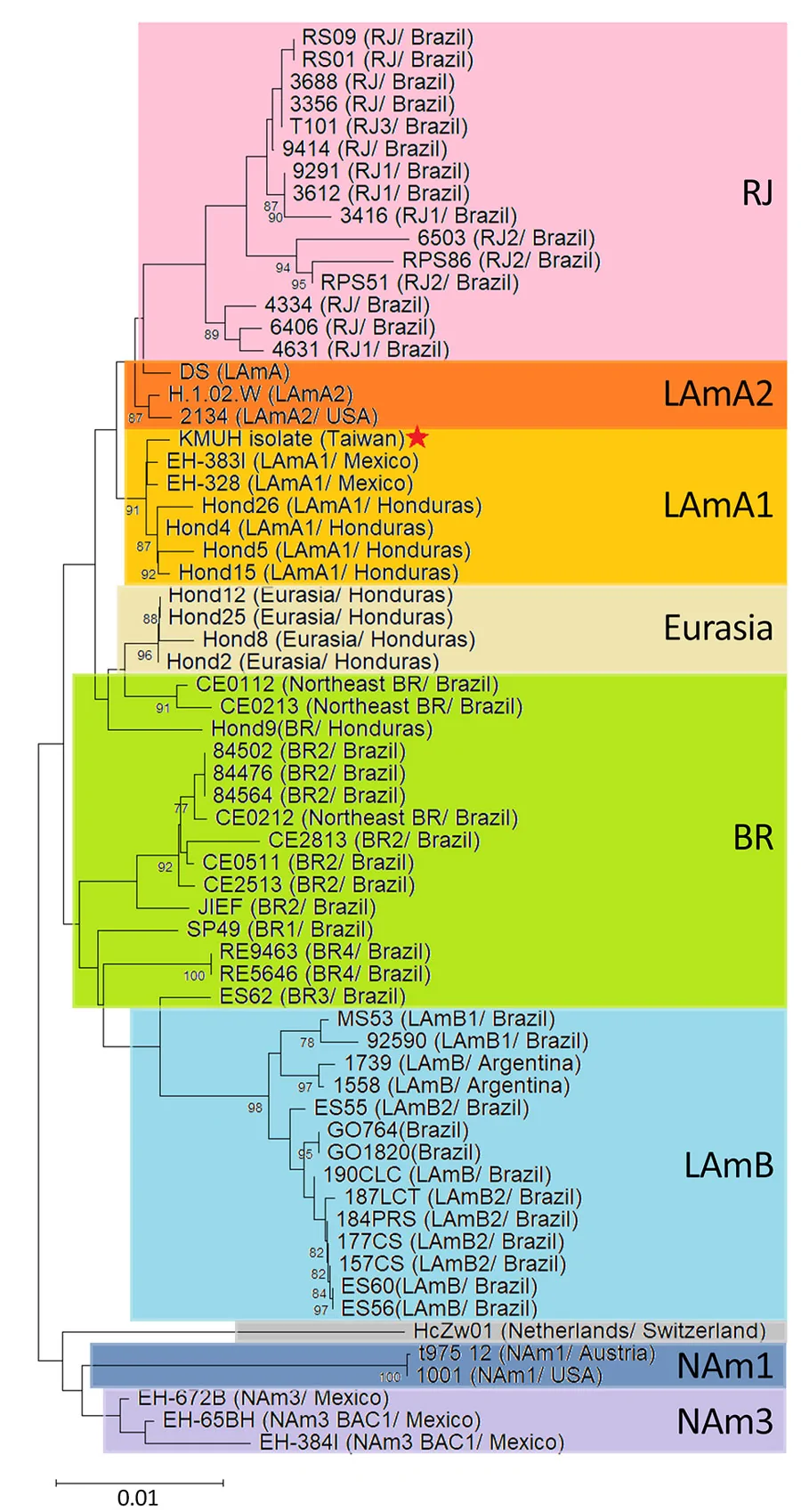
Phylogenetic analysis of *Histoplasma capsulatum* fungi sequences from a case of disseminated histoplasmosis, Taiwan, 2024. Phylogenetic tree was constructed by using the neighbor-joining method on the basis of concatenated multilocus sequence typing analysis of 4 genes (*arf*, *H-anti*, *ole*, and *tub*). Bootstrap support for the relevant internal node was 91% on the basis of 1,000 replicates. Red star indicates isolate sequenced from our study (GenBank accession nos. PX954369 [*arf*], PX954370 [*H-anti*], PX954371 [*ole*], and PX954372 [*tub*]). Scale bar indicates nucleotide substitutions per site. BR, Brazilian; LAmA1, Latin American A1; LAmA2, Latin American A2; LAmB, Latin American B; NAm1, North American 1; NAm3, North American 3; RJ, Rio de Janeiro.

Our findings provide molecular epidemiologic evidence of an unexpected *H. capsulatum* Latin American A1 lineage in Taiwan. Given the increase in histoplasmosis in Taiwan (*1*,*5*), expanded molecular surveillance and environmental sampling are needed to clarify the geographic distribution and transmission ecology of *H. capsulatum* fungi.
